# A major shift of viral and nutritional risk factors affects the hepatocellular carcinoma risk among Ivorian patients: a preliminary report

**DOI:** 10.1186/s13027-015-0013-1

**Published:** 2015-06-30

**Authors:** Alphonsine Kouassi M’Bengue, Moussa Doumbia, Stéphane Romaric Denoman, Djeneba Ngnoh Ouattara, Innocent Adoubi, Pascal Pineau

**Affiliations:** Unit of bacterial and viral serology, Pasteur Institute Ivory Coast - Department of Microbiology, Medical Teaching Félix Houphouet-Boigny University, Abidjan, 01 BPV 166 Ivory Coast; Department of Epidemiology and Clinical survey, Pasteur Institute, Abidjan, 01 BP 490 01 Ivory Coast; Cancer registry-Teaching Hospital of Treichville, Department of Immunology and Cancerology, Medical Teaching Félix Houphouet-Boigny University, Abidjan, 01, BPV 166 Ivory Coast; Unité “Organisation nucléaire et oncogenèse”, INSERM U993, Institut Pasteur, Paris, France

**Keywords:** Aflatoxin B1, Ethnicity, Hepatocellular carcinoma, Hepatitis B virus, Hepatitis C virus, Maize

## Abstract

Hepatocellular carcinoma (HCC) is a major public health concern in Sub-Saharan Africa. Early research in Ivory Coast showed that chronic hepatitis B and aflatoxin B1 exposure were the two most important etiological agents of HCC in the country but, surprisingly, no survey analyzing HCC etiologies has been conducted since decades.

In a preliminary report, we characterized for hepatitis B and C markers 30 consecutive cases of HCC recruited from Abidjan hospitals between June 2011 and December 2012. Nutritional and lifestyle features of patients were analyzed as well. The mean age of the patients was 53 ± 15 years with a sex ratio (M:F = 2.7). HBsAg was the most frequent viral marker in the series (63 %). All HBV isolates belonged to genotype E. With regards to regional standard, anti-HCV reached a very high level (47 %) in the present series. Hepatitis C was more frequent among patients living outside Abidjan (83 vs 23 %, P = 0.009). Patients living in Abidjan were significantly younger than individual living elsewhere in the country (48 ± 14 vs 60 ± 16 years old, P = 0.038) reflecting a possible role for local environmental pollution in tumor progression. Finally, we observed that patients born in Mandé/Gur-speaking regions (North) were younger (48 ± 14 vs 59 ± 15, P = 0.05) and consumed maize more frequently (80 vs 26 %, P = 0.009) than other patients. Interestingly, maize consumption was associated with a trend for aminotransferases elevation (mean = 1.7-1.8 fold, P = 0.06) suggesting a direct hepatic toxicity of this staple food in Ivory Coast. In conclusion, our work indicates that HCC epidemiology underwent recently major drifts in Ivory Coast.

**Electronic supplementary material:**

The online version of this article (doi:10.1186/s13027-015-0013-1) contains supplementary material, which is available to authorized users.

## Background

Early reports from Africa noted the frequent occurrence of hepatocellular carcinoma (HCC) and this disease remains one of the most common cancers on this continent [1]. Chronic infections with hepatitis B virus (HBV) or hepatitis C virus (HCV) are known to be major etiological factors of liver cancer worldwide including Sub-Saharan Africa. Half of incident HCC cases worldwide are still attributable to HBV [2]. Among others, an important risk factor of liver carcinogenesis in Africa is the aflatoxin B1 (AFB1), a mycotoxin known to frequently contaminate staple food in the inter-tropical regions [3].

HBV is a partially double–stranded DNA virus and one of the most important causes of morbidity and mortality worldwide [4]. Globally, it is estimated that about two billion people have been infected with HBV and about 350 million people are chronically infected [5]. Areas with highest HBV prevalence include Eastern Asia and Sub-Saharan Africa, where approximately 10 % of the populations are chronic carriers [6]. In Sub-Saharan Africa, high–risk groups for HBV infection include children born from hepatitis B surface antigen (HBsAg) positive mothers, intravenous drug users, individuals with high-risk sexual behaviors including homosexual men, patients undergoing hemodialysis and health care workers [6–8].

Hepatitis C virus is a single stranded RNA virus that infects 130–210 million people worldwide and at least one third of HCC cases are attributed to HCV infection. There are many geographical variations in HCV chronic carriage due to the heterogeneity of risk factor prevalence between countries. HCV infection usually occurs following direct parenteral exposure to contaminated body fluids, especially blood [9]. High rates of chronic infections have been historically related to inappropriate medical practices as in Egypt or Japan where massive anti-bilharzial prophylactic campaigns led to widespread contaminations [10, 11]. In Sub-Saharan Africa, the prevalence of chronic hepatitis C in the general population is rather low except in selected countries from Central Africa (Cameroun, Rwanda). Contamination with HCV results in chronic infection in 75–80 % of cases and a significant subset of patients evolve toward liver cirrhosis and HCC [12, 13]. The yearly risk of developing HCC ranges from 1 to 5 % after two decades of chronic infection [12]. Overall, the role of HCV in the epidemiology of HCC in Sub-Saharan Africa is rather poorly documented.

Aflatoxins are mycotoxins of the furanocoumarins group that are common staple food contaminants in developing countries. It is estimated that around four billion persons are to some extent chronically exposed to aflatoxins. There are four major aflatoxins (B1, B2, G1 and G2) produced by filamentous fungi of the genus *Aspergillus* [14]. AFB1, the most potent hepatocarcinogenic compound of the family, is converted in human organism by selected isoforms of cytochrome P450 (*e.g. CYP1A2*, *CYP3A4*) in aflatoxin-8,9-epoxide that react with DNA or proteins to form adducts [15]. Aflatoxin M1, the major metabolite commonly found in urine or breast milk of mammals, has ten times less carcinogenic potency than AFB1. Acute toxicity of AFB1 is frequent in animals but is rarely observed in humans except for children. The major symptom of acute aflatoxicosis is the hemorrhagic necrosis of the liver [16]. In areas where exposure to AFB1 is chronic, mutations of *TP53* on the codon 249 (ARG > SER) are found in up to 50 % of cases. These mutations are considered, therefore, as a fingerprint of AFB1 activity [17].

Ivory Coast is located in West Africa along the Gulf of Guinea shores. The country is characterized by the highest environmental diversity in West Africa (mangrove swamps, forests, mountains and savannas) [18]. Population comprises more than 60 linguistic groups that can be regrouped in 4 major ethnicities (Akan and Kru in the South, Mandé and Gur in the North of the country) [19]. Due to the impact of the environment and lifestyle on human liver tumorigenesis, some degree heterogeneity in the natural history of tumorigenesis can be expected in Ivory Coast. Early reports have described the clinical features of HCC in Ivory Coast. In the 1960–70 decades, these tumors were characterized by a very high sex ratio (M:F = 10-20), a younger mean age of onset (42–47 years old), a high rate of associated cirrhosis, and a low socio-economic status of the patients. An instrumental role of the AFB1 was heavily suspected at that time [20–23]. In addition, early molecular analyses have shown that HBV DNA integration played a major role in liver tumorigenesis [24, 25]. More recently, the sex ratio had decreased significantly (M:F = 3) whereas the mean age of tumor onset was stable (47 yo) regarding the previous reports [26]. In the recent period, liver cancer still ranked at the 2nd and 4th position for men and women in Ivory Coast [27].

Viral, nutritional or environmental risk factors (RFs) of HCC in Ivorian patients are suspected to have changed due to the major demographic, economical and environmental evolution that affects Ivorian populations for the last decades. To assess whether the epidemiological bases of liver tumorigenesis drifted during the last decades in Ivory Coast, we set out to evaluate the role played by common RFs in patients with HCC. Preliminary results indicate that substantial changes had occurred in Ivory Coast regarding risk factors of HCC. This situation prompted us to present our data as a warning signal to Public Health authorities.

## Results

### Demographic and lifestyle data

**Fig. 1:**
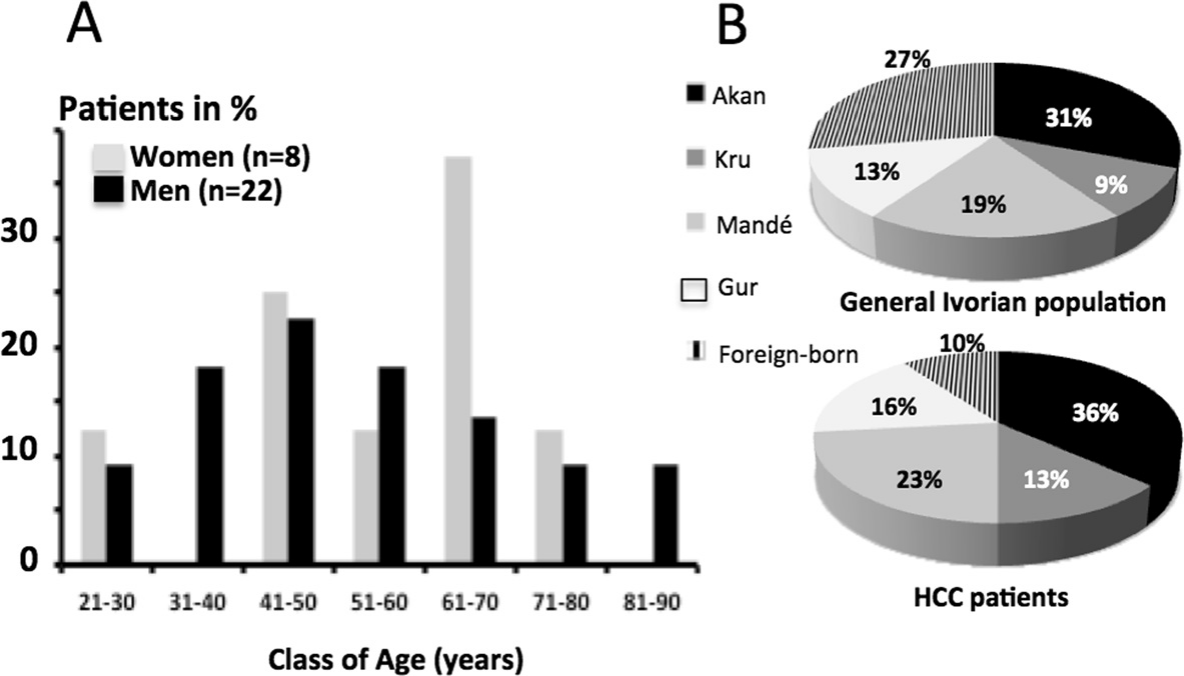
**a**-Distribution of HCC cases, according to age. **b**-Maternal language distribution in patients with HCC. The general linguistic distribution in Ivory Coast was obtained from Biekanh FK [28]

**Fig. 2:**
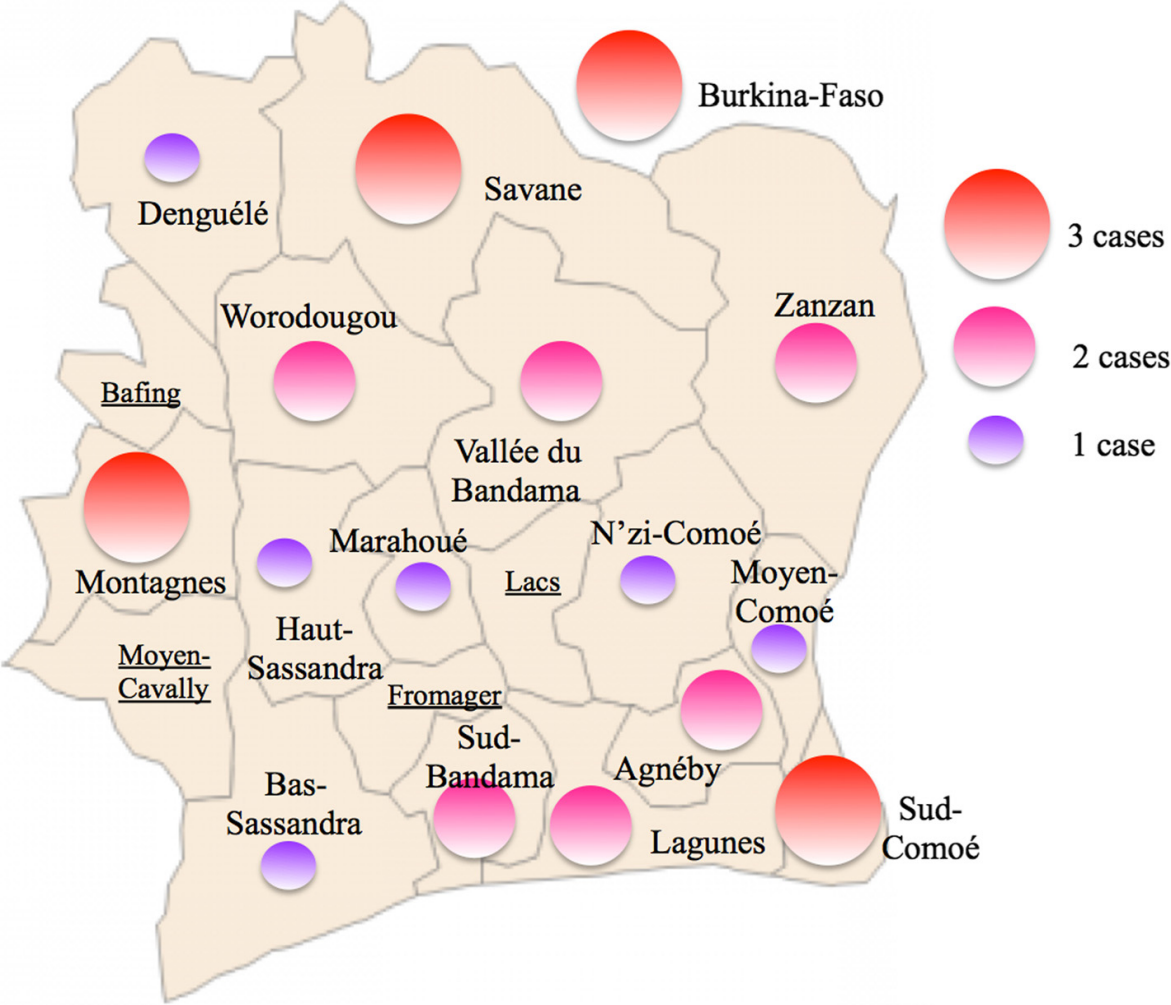
Regions of origin of the 30 patients with HCC. A conspicuous lack of cases is observed in median regions of the country (Bafing, Moyen-Cavally, Fromager and Lacs/Lakes)

Out of 40 patients with HCC who were interviewed during the study period (June 2011-December 2012), 30 (75 %) accepted to participate to the current survey: 22 men and eight women (sex ratio of 2.75, see Table 1). Mean age was 53.7 ± 15.8 years (range 27–82, see Fig. 1a). Overall, 5 (16.7 %) patients lived in a dwelling sheltering more than 2 persons per room, a condition corresponding to low socioeconomic status according to local standards (http://www.ins.ci). Patients were born in the four major ethno-linguistic areas of the country without significant difference regarding the distribution of these groups in the general Ivorian population (see Fig. 1b) [28]. Foreigners were less represented than expected (10 vs 27 %). 15 out of the 19 regions (79 %) of the country were represented among the birthplaces of the patients (see Fig. 2).

**Table 1:** Socio-demographic and clinical features of the 30 patients with HCC

Patients baseline characteristics	
*Socio-demographical features*	
Sex-ratio (M:F)	2.75
Median age, yr (IQR^a^)	53.7 (43–64)
Low socio-economical standard^b^ *n* (%)	5 (16.7)
Living in Abidjan n (%)	18 (60.0)
*Ethnical origin*	
Akan-speaking areas n (%)	11 (36.6)
Gur-speaking areas n (%)	5 16.6)
Kru-speaking areas n (%)	4 (13.3)
Mandé-speaking areas n (%)	7 (23.3)
Foreign born (Burkina Faso) n (%)	3 (10.0)
*Lifestyle features*	
History of heavy drinking n (%)	7 (23.3)
Tobacco use n (%)	3 (10.0)
Cassava consumption n (%)	19 (63.3)
Maize flour consumption n (%)	16 (53.3)
Peanut consumption n (%)	20 (66.7)
*Risk factors of infection*	
Dental treatment n (%)	8 (26.6)
Invasive medical practices^c^ n (%)	11 (36.6)
Ritual scarifications n (%)	12 (40.0)
Sex-transmitted infections n (%)	3 (10.0)
*Clinical features*	
Metabolic disease (Type 2 diabetes, obesity) n (%)	6 (20.0)
Hepatomegaly n (%)	25 (83.3)
Jaundice n (%)	21 (70.0)
Bruises n (%)	7 (23.3)
Portal anastomoses n (%)	5 (16.6)
Liver pain n (%)	20 (66.7)
Ascites n (%)	21 (70.0)
Multi-nodular tumors n (%)	27 (90.0)
ECOG clinical stage**** *n* (%)	
- 1 or 2	8 (26.7)
- 3 or 4	22 (73.3)
*Biochemistry*	
Alpha-feotoprotein >400 ng/mL n (%)	13 (43.3)
Hemoglobin (g/dL ± SD)	10.2 ± 2.0
Prothrombin ratio (% ± SD)	69.3 ± 20.5
ASAT (UI/mL, mean ± SEM)	211 ± 45
ALAT (UI/mL, mean ± SEM)	143 ± 26

^a^IQR = Interquartile range; ^b^6 persons living in <3 rooms-house

^c^: transfusion, surgery, drug dispensation with non disposable syringes

****ECOG = Eastern Cooperative Oncology Group

Lifestyle features commonly associated with an increased risk of HCC were analyzed. Heavy drinking (>200 g ethanol/week for men, >100 g for women) was reported for seven patients (23.3 %) whereas a history of tobacco use was found in only three of them (10 %, see Table 1). Regarding nutritional RFs, major subsets of patients (53.3-66.7 %) commonly consumed peanuts and maize, two commodities known as heavily contaminated with AFB1 in Ivory Coast [29]. Remarkably, peanut consumers were frequently maize flour user as well (70 % of cases) contrasting with habits of patients who do not eat peanut (only 20 % were using maize flour, *P* = 0.018).

Exploring the risks of infection, we observed that 11 (36.6 %) of patients have been subjected to invasive medical practices commonly associated with a high risk of viral contamination in developing countries (blood exposure accidents, blood transfusions, *etc.*…, see Table 1). Traditional scarifications, found in 40 % of cases (n = 12), were present in older patients (60 ± 16 vs 49 ± 13 yo, *P = 0.062*, NS), suggesting that this tradition is maybe currently waning in the country. Finally, metabolic risk factors (type 2 diabetes and obesity) were found in only six patients (20 %). These patients were significantly older than the others (62 ± 4 vs 51 ± 17 y.o., *P* = 0.02).

### Clinico-biological features

The clinical records of the series correspond to very advanced tumors. HCC was multi-nodular in 90 % of the cases; hepatomegaly was present in 83 %, ascites in 66 %, and jaundice in 70 %. The distribution according to the ECOG clinical stages were one patient (3.3 %), seven patients (23.3 %), 16 patients (53.3 %), and six patients (20 %) respectively at stages 1, 2, 3, and 4 (see Table 1) [30].

Concerning laboratory test, AFP was measured above a level 400 UI/mL in 43.3 % of cases. Younger patients (1st quartile) were more frequently high AFP expressers than older ones (86 vs 31 %, *P* = 0.027, see Additional file 1: Figure S1A). No women were displaying AFP above the diagnostic threshold. This difference was statistically significant with regard to males (0 *vs* 52 %, *p* = 0.011, see Additional file 1: Figure S1B). Aminotransferases were determined for 24 patients with abnormalities in all (100 %) cases (Table 1).

### Virological data

**Fig. 3:**
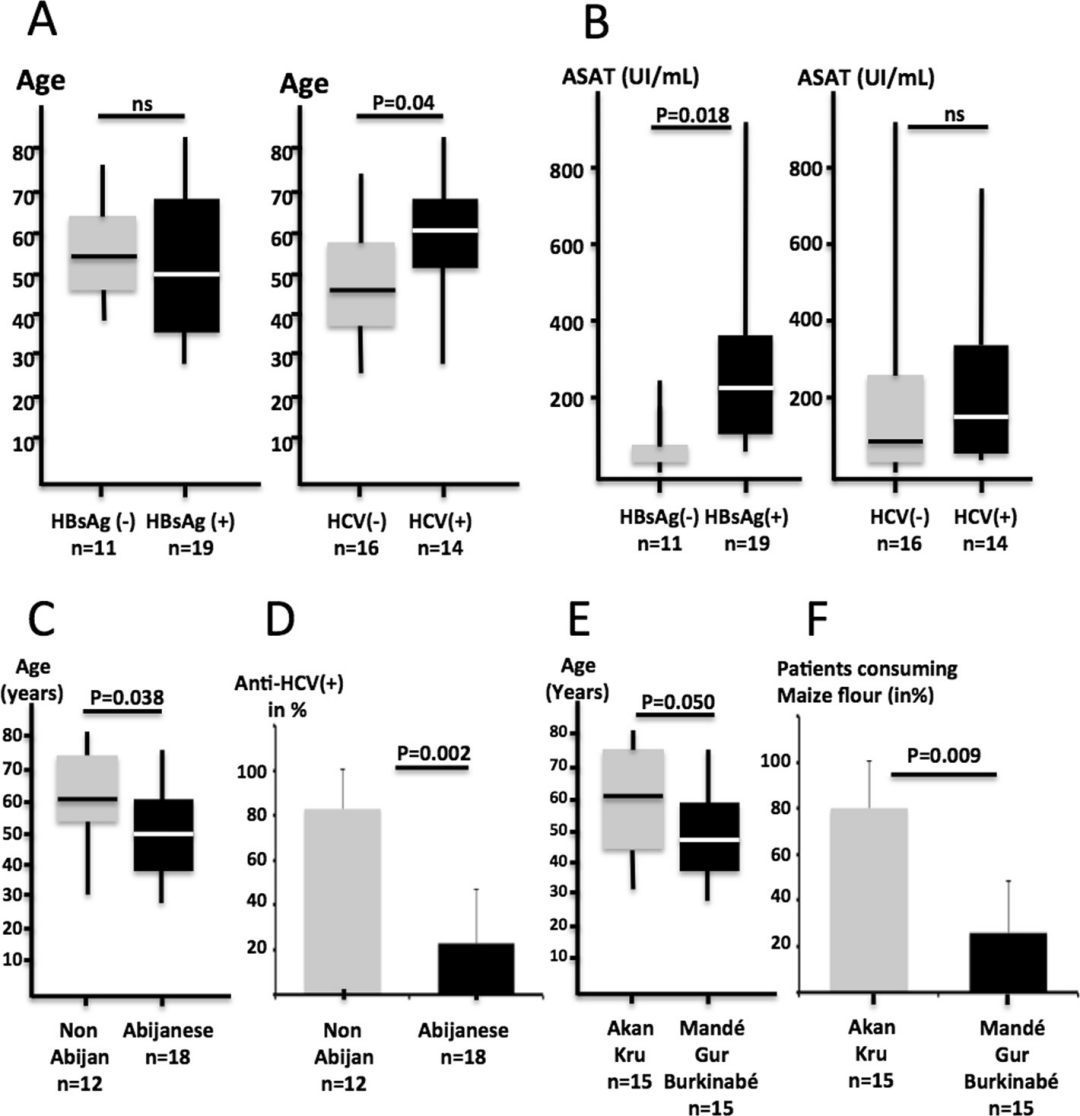
**a**-Anti-HCV(+) patients are older than anti-HCV(−). Age distribution of HBsAg(+) and (−) patients is shown for comparison. **b**-Liver damage is more important in case of chronic infection with HBV (HBsAg(+)) than in the case of chronic hepatitis C. **c**-Patients living in Abidjan are younger than those coming from other regions of the country. **d**-Patients living outside Abidjan are more often anti-HCV carriers. **e**-Patients born in northern regions (Gur and Mandé-speaking) or northern neighboring country (Burkina Faso) are younger than those born in the southern Ivory Coast. **f**-Patients born in the north are more frequently maize consumers

HBsAg and anti-HCV antibodies (Ab) were reactive for 19 (63.3 %) and 14 patients (46.6 %). Overall, 25 (83.3 %) of HCC patients were infected either with HBV or HCV. Co-infection (HBV–HCV) was observed in eight cases (26.6 %, see Table 2). We observed that patients carrying anti-HCV were significantly older than other patients (59 ± 17 *vs* 49 ± 13, *P* = 0.04, Fig. 3a). Overall, 92 % of the patients have been exposed in their lifetime to HBV as shown by the presence of anti-HBcAb. The early HBV antigen (HBeAg), signing active viral replication, was found in five chronic carriers only (26.6 % of chronic carriers) whereas HBV DNA was detected by PCR in 14 cases (73.6 % of HBsAg carriers). All isolates (n = 14/14, 100.0 %) were scored as genotype E. Viral loads in positive samples ranged from 50 and 500 000 copies/mL.

**Table 2:** Viral risk factors in the 30 patients with HCC

Serological and molecular features	n (%)
HBsAg(+)	19 (63.3)
HBeAg(+)	5 (16.6)
anti-HBc(+)	28 (92.5)
anti-hepatitis B immunization	3 (10.0)
anti-HCV (+)	14 (46.6)
Co-infection HBsAg(+)/anti-HCV(+)	8 (26.6)
HBV DNA (+)	14 (46.6)
Genotype E	14/14 (100.0)

n represents the number of positive samples. Percentages are given for the 30 patients except for HBV genotype

Statistical differences were found between HBsAg(+) and other patients. Chronic HBV-infection (HBsAg(+)) was significantly associated with elevation of aminotransferases (210 ± 180 *vs* 97 ± 67 for ASAT and 130 ± 110 *vs* 85 ± 59 for ALAT, P = 0.018 and *P* = 0.024 respectively, Fig. 3b) indicating that, in its terminal phases, chronic infection with HBV is more active than hepatitis C in Ivory Coast. None of the other serological or molecular viral biomarkers were associated with a significant aminotransferases elevation. As expected, aminotransferases levels were normal or sub-normal in nonB and nonC HCC patients, showing significantly lower levels than those observed in infected patients (*e.g.* ALAT, 39 ± 14 *vs* 170 ± 134, *P* = 0.0005, see Additional file 1: Figure S1C).

### Epidemiological correlations

In the current series, the place of living (in or outside of Abidjan) was correlated with specific features. We observed that patients living in Abidjan were significantly younger than patients living elsewhere in the country (48 ± 14 vs 60 ± 16 y.o., *P* = 0.038, Fig. 3c). In addition, Anti-HCV was found in 83 % of outside Abidjan-living patients, a figure much higher than the 23 % rate observed in Abijanese individuals (*P* = 0.002, Fig. 3d). Patients living in Abidjan were also more often positive for HBsAg than others albeit non significantly (61 vs 33 %, ns).

**Fig. 4:**
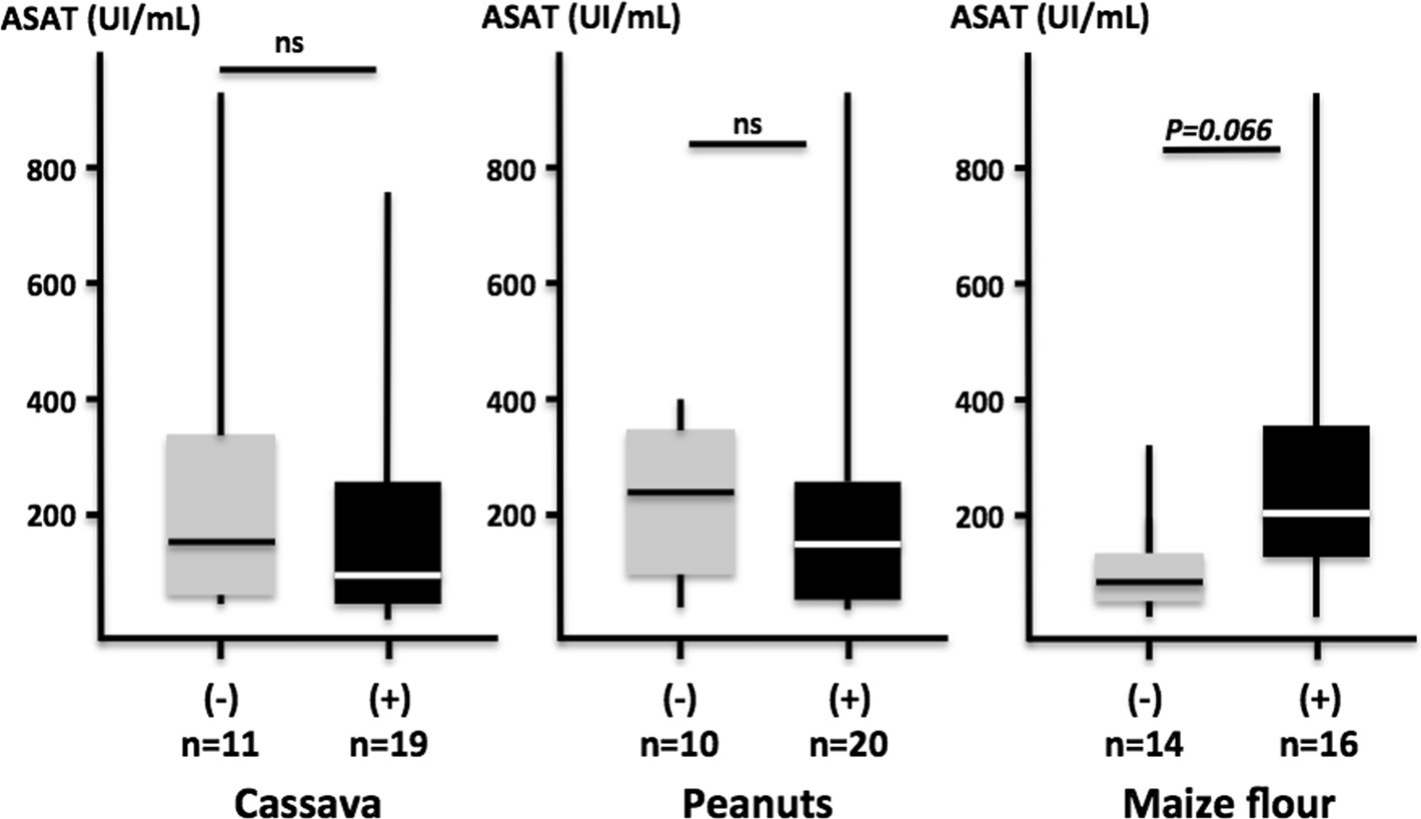
Liver damage as measured by blood aspartate aminotransferase levels after stratification of patients according to staple food consumption. Maize flour appears as more directly hepatotoxic than the other commodities investigated

Likewise ethno-linguistic background was correlated with a specific pattern of HCC in Ivory Coast. Patients born in Mandé- and Gur-speaking regions (North) or in Burkina Faso (a neighboring country on the northern border) were significantly younger than patients born in the South (Akan and Kru) of the country (48 ± 14 vs 59 ± 15, *P* = 0.05, Fig. 3e). Interestingly, these observations correlated with the fact that Mandé and Gur speaking patients were more frequently consumers of maize flour than Akan-Kru patients (80 vs 26 %, P = 0.009, Fig. 3f). Furthermore, we observed that maize flour consumption tended to be associated with significant liver damage as shown by the increase of aminotransferases (ASAT, 296 ± 252 vs 126 ± 92, *P = 0.06*, NS, see Fig. 4). Finally, viral RFs are not evenly distributed in Ivorian HCC patients. Indeed, anti-HCV tended to be less prevalent in patients born in Mandé and Gur-speaking regions than in patients from southern provinces (26 vs 67 %, P *= 0.066*, NS).

## Discussion

As emphasized by several authors, HCC occupies until the current period a position of major importance in cancer epidemiology of Ivory Coast. However, no etiological research had been conducted in the country since almost four decades [22, 27, 31]. Amazingly, the lack of information is rather similar to the five neighboring countries (Liberia, Guinea, Mali, Burkina Faso and Ghana), a situation that prevents the development of appropriate Public Health measures aiming at the reduction of HCC incidence. We present there the first Ivorian pilot study exploring the impact on HCC of the two major viruses responsible of chronic hepatitis virus. Furthermore, we tried to correlate the role of the viruses with nutritional, demographic and ethno-linguistic features historically considered as relevant variables modulating HCC development in the country [20–22].

The mean age of the patients was 53.7 ± 15.8 years (range 27–82) suggesting that HCC onset in the 2010s decade is delayed when compared to the 1970s and 1990s periods where it was diagnosed around the age of 42 and 47 years [26]. The mean age value observed in Ivory Coast is similar to that reported from Mali (52 ± 14 y.o.) or Nigeria (50 ± 17 y.o) but higher than that observed in Burkina Faso (45 ± 12 y.o.) [32–34]. Similarly to a recent observation made in Mali, the incidence peaks were differing between sexes (5th and 7th decades of life in males and females respectively) [35]. The low sex ratio (M:F = 2.75), rather unusual for an African series, may, therefore, partly explain the observed high mean age value. In the present study, 16.7 % (5/30) of patients are living in habitations sheltering more than two persons per room, a promiscuity known to promote intra-familial transmission of hepatitis B and C viruses. These data corroborate the literature stating that HCC has a high incidence in the low-income strata of populations [22, 32]. It is generally considered that the early years of life are particularly important in the history of HCC for Sub-Saharan African patients. Indeed, chronic HBV infection in toddlers and exposure to AFB1 through breast milk are two major factors of HCC development to which African children are exposed massively [36, 37]. Therefore, the origin of patients represents a crucial information to analyze HCC epidemiology in a given African country. In the present series, patients were born in 15 out of the 19 regions of the countries. Examination of the map suggests that patients were coming from two major macro-regions, the northern and the southeastern areas separated by a median territory were HCC appears putatively less frequent (see Fig. 2). This observation should be, of course, confirmed on much larger series of Ivorian patients.

Patients included in the present report were displaying striking differences according to the place where they live. Patients living in Abidjan, regardless of their place of birth or the risk factors they are exposed to, were significantly younger than patients living elsewhere in Ivory Coast. Causes explaining such drastic difference are unknown. The hypothesis of a diagnostic bias with preferentially old patients coming from Ivorian provinces to get treated in Abidjan does not seem plausible. The presence of environmental conditions/agents accelerating the pathological process in a large city as Abidjan (12.7 million habitants in 2011, 4th largest city of Sub-Saharan Africa) represents an alternative hypothesis substantiated by the fact that 4/5 HCC patients without viral RF are living in Abidjan. Remarkably, there is an abundant literature indicating that a preoccupying pollution prevails in Abidjan [38–40]. Another possibility would be a restricted access of the urban dwellers to high quality foodstuffs rich in micronutrients controlling genomic integrity (folates, cobalamine, niacin and zinc) [41]. Finally, although it was not significant, it is worth mentioning that patients from Abidjan were more often HBsAg(+). As a chronic infection with HBV is often the results of newborns or toddlers contamination, it is probable that tumorigenic process began in childhood for these patients, explaining the early appearance of HCC.

The rate of HBsAg(+) HCC patients in the current series (63 %) was similar to those observed historically in the region (55-75 %) [26, 42, 43]. Genotype E of HBV was the only genotype observed in the present series. This genotype, endemic to Western and Central Africa, is known to be dominant (>85 %) in Ivory Coast [44]. It is well known that HBV genotype modulate liver tumorigenesis as it was consistently observed in East Asia where genotype C is more frequently associated with HCC than genotype B [45]. In Africa, genotype E is generally considered as benign when compared to genotype A [46, 47]. Data regarding HCV are far less abundant, but the figure presented therein (46 %) stand well above the corresponding levels published so far in the region (13 and 26 % in Burkina Faso and Mali respectively) [33, 48]. Interestingly, living in Abidjan was associated with a lesser rate of HCV infection. There was no significant difference associated with the risks of infection (dental treatment, ritual scarifications, invasive medical practices) investigated between Abidjan dwellers and other patients. Presence in Abidjan of high-standard medical institutions may explain that the iatrogenic HCV transmission is less frequent in the city than in other provinces of the country. Reciprocally, the relative lack of health professionals in the provinces may conduct patients to consult traditional practitioners less aware of infectious risks associated with the use of non-disposable instruments. Anyhow, the issue of HCV presence at such high levels in the Ivorian population of patients with terminal liver disease deserves further investigation to precisely identify the modes of viral transmission.

Another striking feature of the present series was the younger age of patients born in Mandé- and Gur-speaking areas (North) when compared to Akran/Kru (South). As there was no significant difference regarding ethno-linguistic backgrounds and the presence of chronic hepatitis biomarkers, we turned our attention towards nutritional habits. We observed that patients born in Mandé/Gur-speaking regions were more frequent maize consumers than patients from Akan/Kru regions. Remarkably, our observation that, at variance with peanuts or cassava, maize flour consumption was associated with increased aminotransferases values, suggests that this staple-food may be contaminated by hepatotoxic compounds. In Sub-Saharan Africa, AFB1 is a well-known acute and chronic liver poison. A recently published meta-analysis estimates to 23 % the aflatoxin-related population attributable risk of HCC in the regions where the mycotoxin is abundant [49–51]. The daily level of human exposure to aflatoxin in tropical Africa ranges between 3 and 200 ng/kg [52]. It has been historically suspected that peanuts are the primary responsible for AFB1 intoxication in West Africa. To our knowledge, it is the first time that amino-transferases levels are correlated with maize flour consumption in HCC patients from West Africa. Interestingly, recent reports from Burkina Faso, Mali, Benin and Togo indicate that maize is currently more heavily contaminated by AFB1 than peanuts or autochthonous cereals [36, 53, 54]. Our results confirm these observations and suggests that the attention of Ivorian public health authorities should be now shifted on maize.

## Conclusions

The present pilot study of HCC in Ivory Coast is the first aetiological survey since several decades and produces a somewhat unexpected landscape of this disease in the country. The major information concerns the particularly high rate of infection with HCV (43 %) in Ivorian HCC patients, a level unusual in West Africa, historically dominated by HBV. We observed that this situation is more particularly worrisome outside of Abidjan. Another remarkable feature questioning the distribution of risk factors in the Ivorian population is the young age of two subsets of patients: Mandé/Gur-speaking areas born (North) and Abidjan-living. The young age of Mandé/Gur-speaking patients was plausibly linked to a higher rate of maize consumption, a food known as heavily contaminated by AFB1 but massively introduced in regimens of West Africans in the recent period. The case of Abidjan dwellers was unforeseen but might be related to an easier access to strong alcoholic liquors, or to the important levels of environmental pollution described in the “Lagunes” (lagoons) region where is located the biggest city of Ivory Coast. Due to the small size of the population studied we have to be cautious regarding conclusions, however, a larger scale epidemiological study is now clearly warranted to confirm our observations and to take appropriate measures in the domains of prevention and diagnostics.

## Materials and methods

### Patients

This is a multicenter cross sectional study conducted in Abidjan, Ivory Coast and approved by the Ivorian Ministry of Health Ethics committee. All included patients provided written informed consent. We included all adult patients willing to participate and diagnosed with HCC in one of the four sites identified for the study over the period June 2011 to December 2012. The four recruitment centers were the followings: Cancerology Registry of Treichville Teaching hospital, General Medicine Department of Treichville Teaching hospital, General Medicine Department of Yopougon Teaching hospital, and General Medicine Department of Cocody Teaching hospital. HCC was diagnosed based on clinical criteria, imagery (ultrasound scanning), determination of the tumour marker alpha–foetoprotein (AFP) and biochemical assessment of liver functions (serum aminotransferases). Out of the 30 patients recruited, 23 (77 %) had AFP determination. Socio-demographical data, clinical history and physical examination data were collected using a standardized questionnaire. Blood samples for serological analyses were collected and sent on ice packs to the Institut Pasteur. Sera were stored at −20 °C until assayed.

### HBV and HCV serologies

HBsAg and anti-HCV were assayed using the Architect® kit (Abbott Diagnostics, Wiesbaden, Germany) according to manufacturer’s instruction. In house positive and negative controls sera were introduced in each EIA run for internal quality control.

### HBV DNA extraction

DNA was extracted from 500 μl of serum using the Promega Cat # A1125 DNA purification kit (Madison, WI, USA), according to manufacturer’s instructions, and eluted in 100 μl of buffer.

### Genotyping

A PCR was performed according to the method of Naito *et al.* to select samples containing HBV DNA. Positive PCR was subjected to genotyping assay [55]. A genotyping system based on multiplex-nested PCR using type-specific primers were employed in assigning genotypes A through F based on pre-S1through S genes of the HBV genome. The sequences of PCR primers used in this study are shown in Additional file 2: Table S1. The P1 and S1-2 were universal outer primers. Primer B2 was used as the inner sense primer with a combination of other anti-sense primers for genotypes A, B, and C in a multiplexing system called “Mix A”. Primer B2R was used as the anti-sense inner primer with a combination of sense primers for genotypes D, E and F in a multiplex system called “Mix B”. The genotype-specific primers were relying on sequence conservation within a genotype and poor homology with the sequences derived from other HBV genotypes.

### Real-time PCR quantification of HBV DNA

Viral DNA was purified before quantification. The Nuclisens magnetic extraction protocol (BioMerieux, France) was used to perform this purification: 200–500 μl of plasma and 40 μl of silica were added to 1 ml of lysis buffer; the solution was incubated 10 min at room temperature. Then, it was centrifuged 1 min and the pellet was rinsed with different washing buffers by magnetic stirring in Minimag (BioMerieux). 50 μl DNA were eluted and stored at −20 °C or used immediately in molecular test.

After purification, Viral DNA was quantified by real-time quantitative PCR (qPCR) using 7500 Applied Biosystems. The reaction mixture contained 1XGo-Taq Flexi-buffer (Promega, Germany), 1.25 mM MgCl_2_, Rox dye (Invitrogen, California), 1Unit Go-Taq DNA polymerase, 0.25 mM dNTPs (Promega, Germany), 0.375 μM each primer (hbv305, hbv460), 0.25 μM Probe (15) labeled with carboxyfluorescein (FAM) at the 5’end and with N,N,N,N-tetramethylrhodamine (TAMRA) at the 3’end. The final reaction volume was 20 μl containing 5 μl of extracted viral DNA and 15 μl of PCR-mix. Negative controls were performed with 5 μl of sterile RNase free-water. The amplification was performed according to the following program: 95 °C for 10 min; followed by 40 cycles of amplification at 95 °C for 15 s, 55 °C for 30 s. Fluorescence of FAM liberated from the probe by TaqMan was measured to determine the amplification threshold cycle (Ct), which was the first cycle at which fluorescent emission was 10-fold higher than the standard deviation of the mean baseline emission. HBV-DNA was serial diluted to concentrations of 500.000, 50.000, 50.00, 5.00, 50 and 5 IU/ml, corresponding to 14.10^6^ -14 copies per reaction, respectively (Additional file 3: Table S2).

### Statistical analysis

In univariate analysis, we compared the differences between patient subsets using the Pearson chi2 test or the Fisher exact test. Variables included were: age, gender, HBV genotype, and the virus loads for HBV. All variables with “P” value under 0.05 were scored as statistically significant. Statistical analysis was performed using SPSS11.5 statistical package.

## Additional files

Additional file 1: Figure S1.
**A.** Proportion of Ivorian patients with AFP levels above the diagnostic threshold (400ng/mL) after stratification for age. **B.** Gender difference for diagnostic AFP levels. **C.** Aminotransferase levels in virus-associated and non-viral HCC cases. Additional file 2: Table S1. Sequences of primers used for this study. Additional file 3: Table S2. Clinical linear dynamic range of real time VHB PCR.

## Abbreviations

Ab: Antibody
AFB1: Aflatoxin B1
AFP: Alfa-fetoprotein
Ag: Antigen
ALAT: Alanine aminotransferase
ASAT: Aspartate aminotransferase
HBV: hepatitis B virus
HCC: Hepatocellular carcinoma
HCV: Hepatitis C virus
RF: Risk factor
