# Supplementary material for: A major shift of viral and nutritional risk factors affects the hepatocellular carcinoma risk among Ivorian patients: a preliminary report
Source: Infect Agent Cancer. 2015 Jun 30;10:18. doi: 10.1186/s13027-015-0013-1 (PMC4486136; doi:10.1186/s13027-015-0013-1)

**Legend to the Supplementary Figure**

**Supplementary Figure 1**: **A**. Proportion of Ivorian patients with AFP levels above the diagnostic threshold (400ng/mL) after stratification for age. **B**. Gender difference for diagnostic AFP levels. **C**. Aminotransferase levels in virus-associated and non-viral HCC cases.


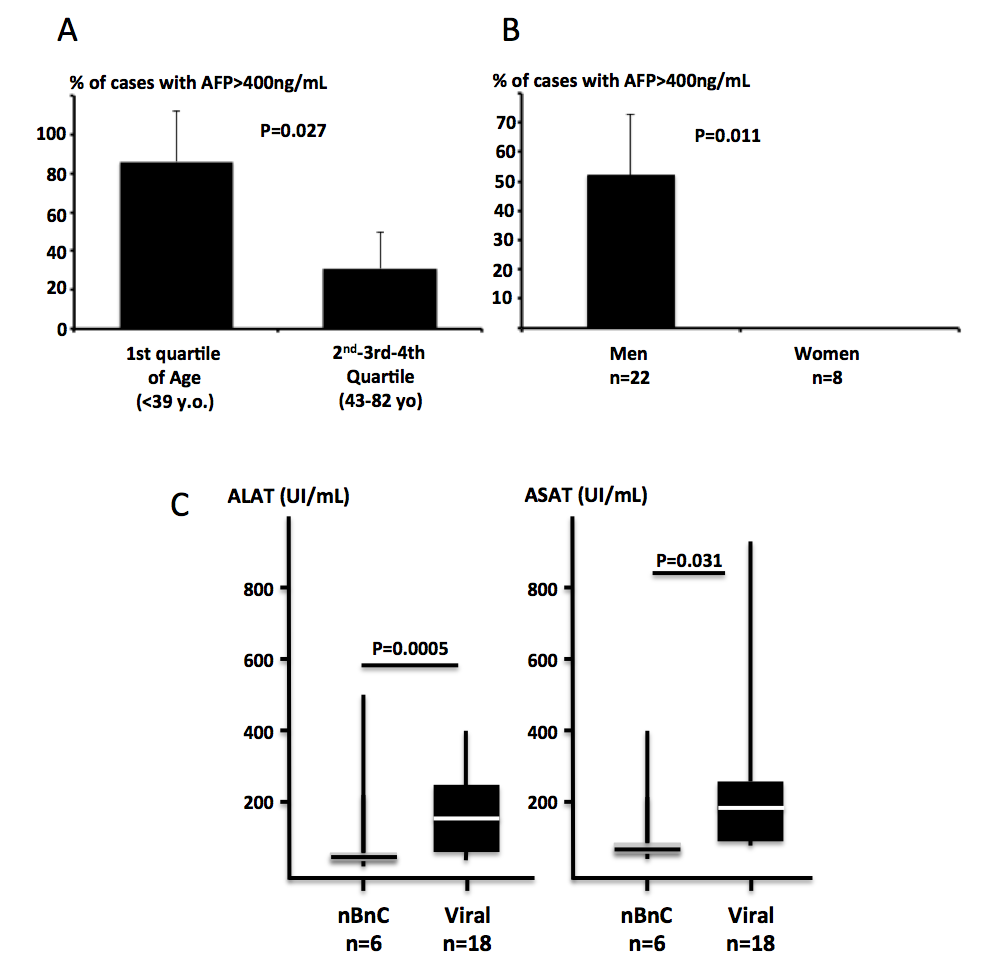

Supplement: Additional file 1: Figure S1. — A. Proportion of Ivorian patients with AFP levels above the diagnostic threshold (400ng/mL) after stratification for age. B. Gender difference for diagnostic AFP levels. C. Aminotransferase levels in virus-associated and non-viral HCC cases. [file 13027_2015_13_MOESM1_ESM.docx]
