# Supplementary material for: A major shift of viral and nutritional risk factors affects the hepatocellular carcinoma risk among Ivorian patients: a preliminary report
Source: Infect Agent Cancer. 2015 Jun 30;10:18. doi: 10.1186/s13027-015-0013-1 (PMC4486136; doi:10.1186/s13027-015-0013-1)
Supplement: Additional file 2: Table S1. — Sequences of primers used for this study. [file 13027_2015_13_MOESM2_ESM.docx]

**Supplemental Table 1**: Sequences of primers used for this study

Primers Sequences Position on HBV DNA

P1 5’-TCA CCA TAT TCT TGG GAA CAA GA-3’ nt 2823-2845

S1-2 5’-CGA ACC ACT GAA CAA ATG GC-3’ nt 685-704

B2 5’-GGC TCM AGT TCM GGA ACA GT-3’ nt 67-86

BA1R 5’- CTC GCG GAG ATT GAC GAG ATG T-3’ nt 113-134

BB1R 5’- GGT CCT AGG AAT CCT GAT GTT G-3’ nt 165-186

BC1R 5’- CAG GTT GGT GAG TGA CTG GAG A-3’ nt 2979-2996

B2R 5’- GGA GGC GGA TYT GCT GGC AA-3’ nt 3078-3097

BD1 5’-GCC AAC AAG GTA GGA GCT -3’ nt 2979-2996

BE1 5’- CAC CAG AAA TCC AGA TTG GGA CCA – 3’ nt 2955-2978

BF1 5’- GYT ACG GTC CAG GGT TAC CA – 3’ nt 3032-3051

Mix A: Type A-68bp, Type B-281 bp, Type C-122 bp;

Mix B: TypeD-119 bp, Type E-167 bp,Type F-97 bp
