# Supplementary material for: A major shift of viral and nutritional risk factors affects the hepatocellular carcinoma risk among Ivorian patients: a preliminary report
Source: Infect Agent Cancer. 2015 Jun 30;10:18. doi: 10.1186/s13027-015-0013-1 (PMC4486136; doi:10.1186/s13027-015-0013-1)
Supplement: Additional file 3: Table S2. — Clinical linear dynamic range of real time VHB PCR. [file 13027_2015_13_MOESM3_ESM.docx]

Supplemental **Table 2:** Clinical linear dynamic range of real time VHB PCR

| **DNA dilution** | **Threshold cycle (Ct)** | **Viral load (IU/ml)** | **copies/ml** |
| --- | --- | --- | --- |
| 10^0^ | 22 | 500000 | 1400000 |
| 10^-1^ | 25 | 50000 | 140000 |
| 10^-2^ | 28 | 5000 | 14000 |
| 10^-3^ | 31 | 500 | 1400 |
| 10^-4^ | 34 | 50 | 140 |
| 10^-5^ | 37 | 5 | 14 |
